# A COMPASS for VESPUCCI: A FAIR Way to Explore the Grapevine Transcriptomic Landscape

**DOI:** 10.3389/fpls.2022.815443

**Published:** 2022-02-24

**Authors:** Marco Moretto, Paolo Sonego, Stefania Pilati, José Tomás Matus, Laura Costantini, Giulia Malacarne, Kristof Engelen

**Affiliations:** ^1^Unit of Computational Biology, Research and Innovation Centre, Fondazione Edmund Mach, San Michele all’Adige, Italy; ^2^Unit of Plant Biology and Physiology, Research and Innovation Centre, Fondazione Edmund Mach, San Michele all’Adige, Italy; ^3^Institute for Integrative Systems Biology (I2SysBio), Universitat de València-CSIC, Paterna, Spain; ^4^Unit of Grapevine Genetics and Breeding, Research and Innovation Centre, Fondazione Edmund Mach, San Michele all’Adige, Italy

**Keywords:** gene expression, grapevine, transcriptomics, compendium, Python, R, FAIR

## Abstract

Successfully integrating transcriptomic experiments is a challenging task with the ultimate goal of analyzing gene expression data in the broader context of all available measurements, all from a single point of access. In its second major release VESPUCCI, the integrated database of gene expression data for grapevine, has been updated to be FAIR-compliant, employing standards and created with open-source technologies. It includes all public grapevine gene expression experiments from both microarray and RNA-seq platforms. Transcriptomic data can be accessed in multiple ways through the newly developed COMPASS GraphQL interface, while the expression values are normalized using different methodologies to flexibly satisfy different analysis requirements. Sample annotations are manually curated and use standard formats and ontologies. The updated version of VESPUCCI provides easy querying and analyzing of integrated grapevine gene expression (meta)data and can be seamlessly embedded in any analysis workflow or tools. VESPUCCI is freely accessible and offers several ways of interaction, depending on the specific goals and purposes and/or user expertise; an overview can be found at https://vespucci.readthedocs.io/.

## Introduction

The problem of data integration concerns merging data coming from several sources while providing the user with a unique way to access and retrieve them. With the advent of high-throughput technologies there has been a considerable expansion in the production of gene expression datasets and while Illumina sequencing has become the *de facto* standard for RNA-seq experiments, microarrays are still widely used (not to mention of historic relevance) and constitute a wealth of public information. Gene expression databases, such as NCBI GEO, SRA or ArrayExpress are data repositories built to store transcriptomic datasets first and foremost, but are not designed to directly combine their measurements. VESPUCCI v1 (Vitis Expression Studies Platform Using COLOMBOS Compendia Instances) is an integrated gene expression database for grapevine (Jaillon et al., 2007; Velasco et al., 2007) originally published in 2016 (Moretto et al., 2016b) that included, at the time, the expression values of 29,090 genes across 1,744 samples measured on 19 platforms and 58 experiments, mainly taken from the GEO (Barrett et al., 2013) and ArrayExpress (Kolesnikov et al., 2015) public databases. Since its publication, it has been shown to be a valuable resource (Adam-Blondon et al., 2016; Fabres et al., 2017) used to deepen our understanding of the role of particular genes of interest (Leida et al., 2016; Malacarne et al., 2016; Pilati et al., 2017; Cheng et al., 2018), as well as a tool for system-wide analysis (Malacarne et al., 2018). VESPUCCI v1 relied on the work done for the COLOMBOS compendium (Moretto et al., 2016a) with some modifications to better adapt it to work with higher eukaryotes, although the original limitations remained. The first implementation limited the extension potential of the interface with new tools, the ability to easily expose the data resources, and the possibility to embed VESPUCCI v1 in other applications or analysis workflow, restricting the interaction mainly to the web application. More conceptual areas for improvement were related to the sample annotation, which used a custom home-made controlled vocabulary to formally describe experimental conditions, and the data normalization, which did not take into account replicated measurement or consider other methodologies beside logratios. Here we present a new major release of VESPUCCI (Vitis Expression Studies Platform Using COMMAND>_ Compendia Instances), named v2, that includes nearly all grapevine gene expression datasets available in public repositories up to December 2020. It is designed to overcome the limitations of the previous version and has been built from the ground up in order to be compliant with FAIR principles (Wilkinson et al., 2016). The multi-tier architecture implemented for v2, which separates the compendium (VESPUCCI) from the programmatic interface (COMPASS), as well as the structured sample annotation based on ontologies, and the employment of different normalization methods, all improve the accessibility, reproducibility, and interoperability of the resource.

## Method

### Construction Workflow

**FIGURE 1:**
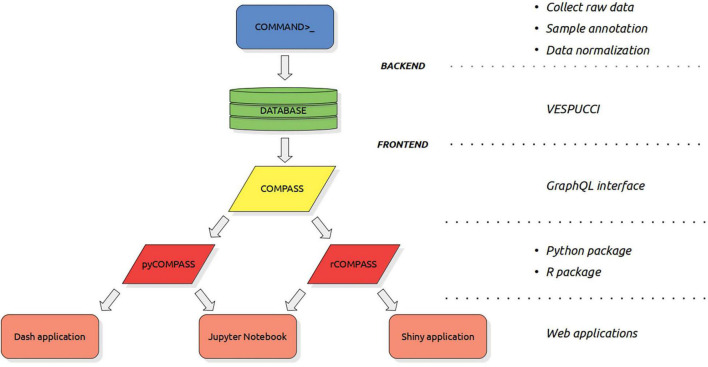
The complete hierarchy of tools and interfaces used to create, query and expose VESPUCCI. From top to bottom: COMMAND>_, that is the backend software developed to create gene expression compendia. COMMAND>_’s main goals are to simplify the process of collecting raw data, annotate sample conditions and normalize experiments. VESPUCCI is the grapevine gene expression compendium built using COMMAND>_. COMPASS is the frontend, that is the main interface to access VESPUCCI. It is a GraphQL interface that allows querying every part of the VESPUCCI data model. pyCOMPASS and rCOMPASS are the two high-level packages (for Python and R, respectively) built on top of the COMPASS interface that simplify the access and analysis of VESPUCCI’s data. Last layer is composed of all the applications that rely on these packages such as GUI applications (like Dash or Shiny applications) as well as analysis workflows such as Jupyter Notebook and R markdown that further simplify the interactions with the database.

The construction of a compendium such as VESPUCCI v2 is a complex process composed of three main steps (see Figure 1). The first one is raw data collection and it has been performed using the COMMAND>_ backend. This part has been extensively described in Moretto et al. (2019). The other two steps are sample annotation and data normalization. Sample annotation refers to the operation of describing sample conditions using ontology terms. Data normalization requires the definition of the experimental design, in which samples are grouped based on the experimental conditions and the fact that may represent biological or technical replicates, along with the kind of normalization to perform, such as LIMMA v3.22.7 (Ritchie et al., 2015) logratio normalization or TPM (Transcript Per Million) (Wagner et al., 2012).

### Annotation System

**FIGURE 2:**
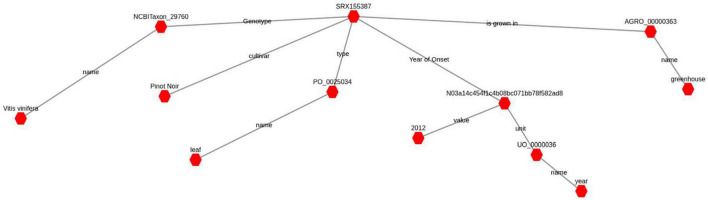
A complete RDF-graph that represents the condition annotation for sample with accession number SRX155387. The red hexagons represent subjects or objects, while gray lines represent predicates connecting them. An object in a triple can be the subject of another triple, in this way all triples contribute to build the complete graph. A blank node, here represented with an alphanumeric ID, serves the purpose of connecting different concepts together, one subordinate to another. In this example the RDF-graph states that the sample SRX155387 is a leaf of a *Vitis vinifera* (cv. Pinot noir) grown in a greenhouse and collected in the year 2012.

Sample annotation has been carried out employing ontologies terms and RDF^1^ (Resource Description Framework). RDF data model is a general method for describing data by defining relationships between entities. RDF is a W3C (World Wide Web Consortium) standard used to describe information. Each RDF statement is a three-part structure consisting of a subject, a predicate and an object. Each RDF term is either a sample or gene ID, an ontological term or a literal, either string or number. A term could also be a special kind of ontological term, called Blank node, that is used to connect different triples and make more expressive statements about specific conditions. Several ontologies have been used to annotate samples and genes, both generic, such as Nci thesaurus (Ceusters et al., 2005) or Dbpedia^2^, and plant specific ones, such as the Plant ontology (Jaiswal et al., 2005) or the Plant trait ontology (Arnaud et al., 2012). SPARQL^3^ (SPARQL Protocol and RDF Query Language) is the query language used to navigate relationships in RDF graph data (see Figure 2) through graph pattern matching, by combining simple patterns into more complex ones allowing the exploration of elaborate relationships in the data.

### Data Normalization

**FIGURE 3:**
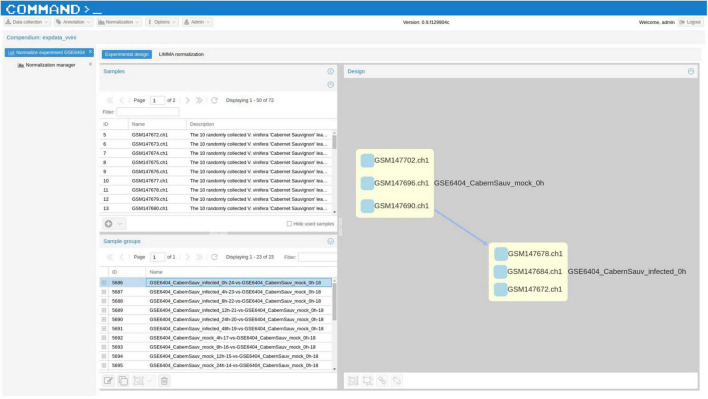
A screenshot of COMMAND>_, the web application employed to build VESPUCCI. This screenshot shows the “Experimental design” tool used to define contrasts between samples for a specific experiment. The top left grid shows the list of all experiments’ samples, while the bottom left grid shows what we called “sample groups,” that is groups of samples that will produce one measurement for each gene. In this case the LIMMA normalization will be applied, thus one group of samples will be arranged as a condition contrast. The sample group displayed in the left diagram represents the replicated samples for the two conditions named GSE6404_CabernSauv_mock_0h and GSE6404_CabernSauv_infected_0h. In the right panel the direction of the arrow indicates how the contrasts should be calculated, that is from the reference condition GSE6404_CabernSauv_mock_0h to the test condition GSE6404_CabernSauv_infected_0h.

**FIGURE 4:**
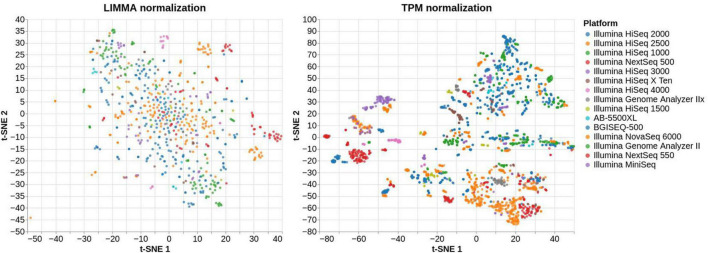
t-SNE plot of the same RNA-seq samples normalized using LIMMA, on the left-hand side, and TPM, on the right-hand side. The right plot shows that a great portion of samples belonging to the same platform are clustered together, meaning that their expression profiles are mostly platform-dependent. On the other hand, the left plot is more scattered and does not show the same pattern of clusters suggesting that most of the batch-effects have been removed.

VESPUCCI v1 originally offered only one normalized matrix using the method developed for the COLOMBOS bacterial compendia (Moretto et al., 2016a) based on “sample contrasts,” i.e., logratios between a test and a reference sample. With the latest update VESPUCCI v2 provides a TPM (Wagner et al., 2012) normalized matrix for RNA-seq data as well as logratios calculated using the R (v3.1.1) package LIMMA v3.22.7 (Ritchie et al., 2015) for both RNA-seq, through the VOOM normalization (Law et al., 2014), and microarray experiments containing biological replicates. The original normalized data matrix, referred to as “legacy,” is still available under the new infrastructure but was not updated with recent experiments. The normalization process is composed of several steps. Once a normalization technique is decided for a specific experiment, the experimental design gets defined by arranging samples in groups of one or more samples. Each sample group will result in one measurement per gene and the way in which samples are grouped and the measurement is calculated depends on the chosen normalization. For example, in the case of TPM normalization each group will be composed of a single sample, since the normalized values get calculated using only the raw data of each sample independently. For LIMMA logratios, instead, one sample group is composed of at least 4 samples, 2 replicates for the reference condition and 2 replicates for the test conditions from which a contrast is calculated (see Figure 3). Comparison between the two normalization methods (Abbas-Aghababazadeh et al., 2018) highlights the impossibility of the TPM normalization to remove platform specificity, while LIMMA successfully removes most of the batch-effects (see Figure 4).

## Results

### The Compendium

**FIGURE 5:**
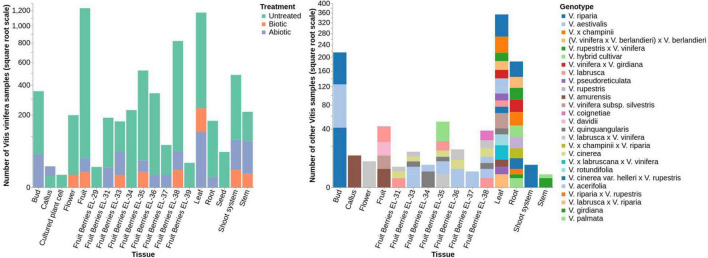
Bar plots of VESPUCCI v2 sample distributions based on their condition annotation in *Vitis vinifera* **(left)** and non-vinifera **(right)** experiments. Samples are divided by tissues (*X*-axis) and their abundances in square root scale (*Y*-axis). In the left plot, different colors are used to denote untreated samples, biotic-treated samples and abiotic-treated samples. In the right plot, colors are used to differentiate between different *Vitis* species, subspecies and cross species. The great majority of samples (47%) come from *Vitis vinifera* untreated fruit samples taken at different developmental stages. Non-vinifera species and hybrids samples represent 13% of the dataset while nearly 9% of the total are stress-related (2.5% being fruit) and 31% are *Vitis vinifera* untreated non-fruit samples.

The VESPUCCI v2 compendium is a comprehensive database of nearly all transcriptomic experiments performed on grapevines during the last 15 years. It contains 3,682 microarray and 3,598 RNA-seq samples across 271 experiments collected until December 2020. VESPUCCI v2 can be thought of as a massive gene expression matrix with rows representing the nearly 29,000 grapevine genes and columns representing biological conditions. The experiments measure expression values coming from 199 different *Vitis* genotypes, 47 different tissues and 31 developmental stages; concerning experimental conditions, 70 different abiotic and 21 different biotic treatments are represented. Figure 5 shows the overall composition of VESPUCCI v2 based on the sample annotation. Gene expression values come from 15 different technological platforms, 5 microarrays and 10 sequencers (see Table 1). Regarding the RNA-seq experiments, the Illumina HiSeq 2000 and 2500 are the most used platforms, while for microarrays the NimbleGen *Vitis vinifera* array platform is both the most used and the one that is able to measure the great majority of genes. This is due to the fact that Nimblegen probes were designed using the CRIBI v1 gene annotation, the same used by VESPUCCI.

**TABLE 1:** Complete list of the technological platforms used to measure gene expression in VESPUCCI.

Platform name	Platform type	LIMMA contrasts	TPM samples	Measureable genes	Measureable probes
NimbleGen	Microarray	515	0	28,017	139,363
Affymetrix GrapeGen		465	0	14,926	264,387
INRA oligo array 15K		20	0	6,583	16,416
Agilent Grape Oligo		22	0	19,250	62,976
GrapeArray 1.2		18	0	8,872	24,676
NextSeq 550	RNA-seq	5	18	29,090	0
NextSeq 500		64	561	29,090	0
Illumina NovaSeq 6000		7	78	29,090	0
Illumina HiSeq 4000		24	158	29,090	0
Illumina HiSeq 2500		230	1,044	29,090	0
Illumina HiSeq 2000		244	1,317	29,090	0
Illumina HiSeq 1000		96	544	29,090	0
Illumina Genome Analyzer IIx		16	57	29,090	0
Illumina Genome Analyzer		5	37	29,090	0
HiSeq X Ten		24	144	29,090	0

*Each row contains the platform name and its type, either microarray or RNA-seq, the total number of contrasts calculated using the LIMMA package, the total number of TPM-normalized samples (for RNA-seq only), the number of measurable genes for that platform (for RNA-seq this number is always the maximum number of genes, 29,090) and the number of probes in each of the microarray platforms.*

### The COMPASS Interface

To expose the VESPUCCI v2 database we developed a front-end API based on GraphQL^4^ technology using Django^5^ v1.11.7 and Graphene^6^ v2.1.3. We refer to this first layer to directly access the VESPUCCI v2 database (and eventually any compendium built using the COMMAND>_ technology) as COMPASS (COMpendia Programmatic Access Support Software). COMPASS presents a complete set of functionalities to query every part of the data model. The basic entity around which most of the analysis revolves is what we call modules. In brief a module is a subset of the whole gene expression matrix. Thus, a module is composed of gene ids as rows, condition (or sample) ids as columns and expression values. COMPASS offers several tools to analyze, extend or modify modules, manually or by relying on co-expression. Together with tools that handle module’s data, COMPASS offers several ways to retrieve information from metadata, such as the SPARQL query language, the module’s summary description, and the enrichment tool that uses both sample and gene annotations. COMPASS can also be used for the retrieval of raw sample data, to be processed and analyzed by users with third party tools. On top of COMPASS we developed two packages, pyCOMPASS and rCOMPASS for the Python and R languages, respectively (see Figure 1 and Supplementary Table 1). The two packages are easily embeddable in any analysis workflow or other applications and provide an easier way to programmatically access the compendium (see section “Data and Code Availability”). Finally, we implemented a custom web application using Dash on top of pyCOMPASS to provide the basic functionalities available in the original v1 application and to show the benefit of a multi-tier architecture that separates the data layer from the presentation and logic layer. As the GUI does not require programming experience, it can be a useful feature to those who are not familiar with it, as well as for quick access to the data. Using the programmatic interface is usually the preferred, most transparent, and most powerful way to access VESPUCCI v2 (query requests and responses are wrapped with language specific objects and functions). Moreover, using one of the two packages within an interactive environment such as a Jupyter Notebook or RStudio is beneficial for the reproducibility of the analysis.

### A Formal and Expressive Paradigm for Sample Condition Annotation

Beside the numerical data representing the gene expression values measured across the different conditions, VESPUCCI v2 provides structured, manually curated metadata. Sample annotation has required most of the human intervention because of the need for interpretation of -often cryptic- sample descriptions. We adopted the RDF data model to describe information since it ensures enough flexibility to describe samples with different levels of expressiveness coping with any kind of complex experimental conditions. For instance, we can formally describe samples for which treatment and collection times occur at different stages. Moreover, each ontology term used to describe samples brings the whole ontology structure with it, providing the possibility to reason about the implicit relationships between samples to make them explicit. For instance, every measurement has been labeled with the corresponding unit of measurement using the UO (Unit of Measurement Ontology), allowing for conversion of one measurement unit into another (providing makes sense), such as days in hours or molarity to mg/ml. RDF triples can be naturally thought of as a graph (see Figure 2) where subjects and objects are connected through predicates. Each graph thus represents the complete condition annotation for a given sample and, as any RDF-graph, it can be queried using SPARQL, the W3C standard language to query RDF data. We implemented a query abstraction layer in order to allow SPARQL queries to be executed within the COMPASS interface and retrieve both samples and genes.

### Differences Between Logratio and Transcript per Million Data Normalization

VESPUCCI v2 provides data normalized with two different approaches: logratios for both microarray and RNA-seq experiments, through the LIMMA (Ritchie et al., 2015) and “legacy” normalizations on one side and TPM values (Wagner et al., 2012), for RNA-seq only, on the other side. While the former offers a less trivial interpretation of the data since it represents a ratio between values measured in two different conditions, it is better suited for comparing samples across different conditions while TPM, despite being more intuitive on the biological interpretation, should be used with caution when comparing samples with very different total mRNA levels or analyzed in different laboratories (Dillies et al., 2013; Zhao et al., 2020). Figure 4 shows a comparison between the LIMMA and TPM normalization methods for the same samples in VESPUCCI v2 using t-SNE for dimensionality reduction. While LIMMA does not reveal evident groups of samples, TPM shows how samples get clustered together based on the platform they belong to. This is due to the technical differences during sample preparation for the different platforms that will not get removed with a TPM normalization and are in fact clearly visible. This should be taken into consideration when using TPM to compare samples coming from different platforms and experiments since most of the variation observed might be due to technical differences instead of biological ones. We thus recommend limiting the usage of TPM normalized data in VESPUCCI v2 to same-experiment samples when performing comparisons. LIMMA, on the other hand, is suitable for comparison across different platforms and experiments since most of the technical variability gets removed by the ratio-based normalization that is always performed between samples coming from the same batch (Luo et al., 2010).

### Comparison Between VESPUCCI v1 and v2

The differences between VESPUCCI v1 and v2 affect all of the features presented above, i.e., the database content, the interface, the sample annotation, and the data normalization (see Supplementary Table 2). First of all, v2 represents a content update over v1 and includes the transcriptomic datasets available up to December 2020. Major contributions from recent experiments are due to RNA-seq data, performed on non-vinifera or hybrid *Vitis* species, and stress experiments (see Supplementary Figure 1). Originally, the database, the query system, and the presentation layer were all combined in a monolithic graphical application. The v2 architecture instead is based on decoupling the actual compendium data (VESPUCCI) from the presentation layer, i.e., the COMPASS interface, allowing the creation of a hierarchy of tools on top of that. The sample annotation has always been the area for which most of the human intervention was required. For v1, we created a custom controlled vocabulary to describe the sample conditions, while for v2 we decided to rely as much as possible on existing ontologies as well as a standardized data model as RDF, simplifying the annotation process and allowing the exploitation of existing tools and resources. As for data normalization, v1 provided only logratios between one test and one reference sample, while with the adoption of the LIMMA package for v2, we were able to exploit replicated measurements for both microarray and RNA-seq using the same software.

### Use Cases

**FIGURE 6:**
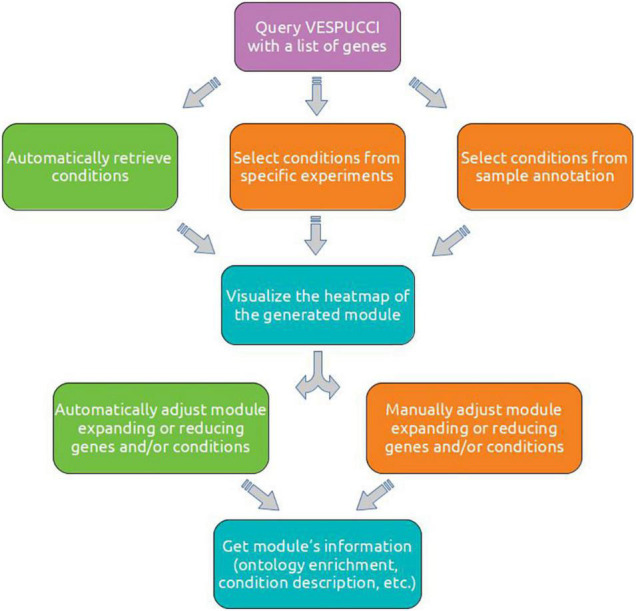
A diagram of the typical steps involved in the analysis of a set of genes of interest. After having retrieved a list of genes, a module can be created automatically or by manually specifying a set of conditions using experiment’s ID or sample annotation. A module can be visually inspected using the clustered heatmap to decide how to refine the module composition by adding or removing either genes or conditions (or both). These expansion and reduction steps can be performed manually or automatically. The refined module can then be inspected using aggregated information such as module description and ontology terms enrichment.

To better illustrate the details of VESPUCCI and to show the characteristics of reproducibility that the use of a programmatic interface and an interactive language provide, we performed different analyses employing pyCOMPASS and Jupyter Notebook through the Google Colab infrastructure^7^. Figure 6 depicts the typical analysis workflow to investigate a set of genes of interest and obtain insights about their putative role based on the conditions in which they are modulated. Detailed information as well as the complete code is available in Supplementary Material as well as in the corresponding documentation (see section “Data and Code Availability”). The first one starts with a short list of genes putatively involved in pollen development. VESPUCCI creates a module that gets expanded with more conditions and genes with the same putative function. The second use case aims to characterize a grapevine gene family and identify those members that show tissue-specific expression and modulation upon biotic stresses. The last use case explores a high number of target genes modulated by the MYB14 transcription factor obtained in Orduña et al. (2022). This set of MYB14-bound genes was filtered according to the TF-binding position in: (i) upstream-bound (up to −3 kb from each transcription start site) and (ii) gene body and downstream-bound (coding sequence and untranslated regions, up to 2 kb from the end of each gene feature). Here, we linked their tissue specificity to functional classes using Gene Ontology enrichment analysis.

## Discussion

During the last decade, several resources for plant transcriptomic data analysis and visualization have been created, such as TomExpress (Zouine et al., 2017), a database of RNA-seq data to visualize, cluster and create correlation networks of expression data for tomato, and Genevestigator (Hruz et al., 2008), a commercial database and toolset for gene expression data for Arabidopsis and other plant species. Several grapevine specific resources have also been developed, such as VitisCyc (Naithani et al., 2014), a knowledge base of metabolic pathways, VTCdb (Wong et al., 2013) and VTC-Agg (Wong, 2020), databases of gene co-expression networks, NES2RA (Pilati et al., 2021), a tool to expand gene networks based on co-expression, Grape eFP Browser (Sullivan et al., 2019) and the Vitis visualization platform VitViz^8^, two tools for visualizing gene expression changes across various plant tissues, miRVIT (Chitarra et al., 2018), a database of miRNA candidates, Grape-RNA (Wang et al., 2020), a database of RNA-seq data, BIOWINE (Pulvirenti et al., 2015), a knowledge base for functional analysis, VitisNet (Grimplet et al., 2009), a knowledge base of molecular networks, as well as VESPUCCI’s original v1 version (Moretto et al., 2016b; see Supplementary Table 3). Some of them are no longer available, require a login to access or present a GUI as the sole mode of interaction limiting the access to both data and tools. The best case-scenario, in terms of interoperability and ensured availability, is to bulk download the entire dataset, as was possible for VESPUCCI v1. Despite the features provided by the GUI, the v1 was limited by the tools offered by the interface in terms of analysis possibilities. By decoupling data (VESPUCCI) from the programmatic interface (COMPASS) in the current v2 version and making all software open source it is easier to create new analysis tools, embed the data in third party services or use them in any analysis workflow, enhancing reproducibility of results and interoperability of different resources. The updated version of the VESPUCCI database integrates nearly all available microarray and RNA-seq transcriptomic experiments, raising the total number of samples to more than 7,200, a 200% increase in quantity compared to the previous version, with most of the new samples coming from RNA-seq platforms. Despite the great number of microarray samples available, it is easy to foresee only RNA-seq in the future of grapevine transcriptomic experiments. In the last years only RNA-seq experiments have been performed, moreover, some microarray platforms are no longer available and above all the advantages of RNA-seq outperform the ones from microarray, like the possibility to measure all genes without being bound to a predefined number of probes as well as the sequence information and the possibility to increase the quality of the results with a higher quality genome to be used for alignment. The sample annotation for the v2 relies on existing standard tools and resources such as ontologies and RDF. There are multiple advantages in respect to the v1. First of all we do not have to develop and maintain a non-standard controlled vocabulary. Second, by using standardized resources we can adopt an ecosystem of other tools such as SPARQL or semantic reasoners based on such standards. Over the years we have witnessed the rise of several resources employing Semantic Web standards (such as RDF) for the life sciences like Bio2RDF (Dumontier et al., 2014), Linked Life Data^9^, the NBDC RDF Portal (Kawashima et al., 2018), the EBI RDF Platform (Jupp et al., 2014) and Uniprot (The UniProt Consortium, 2021) SPARQL endpoint^10^. Most of these expose an RDF triplestore containing newly defined Classes and Properties using the Web Ontology Language (OWL) or RDF-Schema to provide a rich description of RDF triples. Since VESPUCCI is primarily an integrated gene expression database focused on numerical values, a complete sample annotation is a necessary condition for a correct interpretation of gene expression patterns. Nevertheless, it is not the main feature and despite the use of RDF it is not a triplestore as the aforementioned resources. We employed RDF solely as a graph data model in order to have the flexibility needed to describe different experimental conditions that would not be possible with tabular format and to be able to exploit third party tools that use the same standard. Moreover, we decided to rely as much as possible on existing ontological terms without the need to define new terms. This has been done to prevent the increasing heterogeneity and discrepancy shown in the Life Sciences Linked Open Data (Kamdar and Musen, 2021). The last improvement of v2 over v1 concerns data normalization. In v1 only one normalization method was available, while in v2 different normalization methods are offered to serve different analysis purposes such as studying patterns of gene expression or their expression levels. Recently we have witnessed an increasing awareness of the need to promote the use of shared resources and international standards to fulfill the view of the FAIR principles. Within the grapevine community several points have emerged as the most pressing ones to be addressed (Adam-Blondon et al., 2016), such as having a single entry point for resources, the manual curation of data and metadata, the use of widely adopted standards, the use of ontologies and controlled vocabularies, the possibility to access data through web services and API’s, and the possibility to integrate analysis workflows. Together they aim to implement the four principles of FAIRness: findability, accessibility, interoperability and reusability of data. VESPUCCI v2 attempts to fulfill this vision by providing a single point of access to the database via a web server GraphQL interface, a manually curated annotation of experimental conditions using ontologies and RDF as data models, and software packages for the two most widely used programming languages in data analysis, Python and R, to enhance the interoperability of VESPUCCI v2 with other resources and tools. The changes made in the backend technology allow for more frequent updates and the coexistence of different versions for the same normalization technique as well as the implementation of more normalization techniques starting from the same raw data. In this regard, future versions of VESPUCCI will include, together with newly available experiments, SNP marker information and new normalization techniques, as well as using the upcoming VCost.v4 gene annotation alongside the current versions. The reason why the VCost.v3 (Canaguier et al., 2017) gene annotation has not been used for the current version of VESPUCCI is due to the benefit to cost ratio. In addition to the updates on the backend, we are also working toward the improvement of the user experience by extending the current number of tools and interfaces built on top of COMPASS. These enhancements might come from the creation of programmatic interfaces for other languages besides Python and R, the creation of standardized workflow for specific analysis, and the use of compilers for other DSLs (Domain Specific Languages) toward GraphQL.

## Data and Code Availability

**Table:** 

Resource	URL
VESPUCCI v2 use cases and documentation	https://vespucci.readthedocs.io
COMPASS GraphQL interface	http://compass.fmach.it/graphql
COMPASS documentation	https://compass-.readthedocs.io
pyCOMPASS documentation	https://pycompass.readthedocs.io
rCOMPASS documentation	https://onertipaday.github.io/rcompass
DASH GUI application documentation	https://compass-.readthedocs.io/en/latest/dashcompass.html
pyCOMPASS package download	https://pypi.org/project/pyCOMPASS/
rCOMPASS package download	https://github.com/onertipaday/rcompass
DASH GUI application	http://compass.fmach.it/dashcompass
COMPASS source code	https://github.com/marcomoretto/compass
pyCOMPASS source code	https://github.com/marcomoretto/pyCOMPASS
rCOMPASS source code	https://onertipaday.github.io/rcompass/
DASH GUI application source code	https://github.com/marcomoretto/dashcompass

The complete list of packages (together with their version number) used for the development of these tools is available in the requirements.txt file in each of the GitHub repositories.

## Data Availability Statement

The original contributions presented in the study are included in the article/Supplementary Material, further inquiries can be directed to the corresponding author.

## Author Contributions

MM and PS conceived the work, implemented the procedures, analyzed the data, wrote the manuscript, and collected and processed the data. MM, PS, SP, LC, and GM performed the meta-data annotation. MM, PS, and KE beta-tested the application. SP, JTM, LC, and GM provided the use cases. MM, PS, SP, JTM, LC, GM, and KE revised and edited the manuscript. All authors contributed to the article and approved the submitted version.

## Conflict of Interest

The authors declare that the research was conducted in the absence of any commercial or financial relationships that could be construed as a potential conflict of interest.

## Publisher’s Note

All claims expressed in this article are solely those of the authors and do not necessarily represent those of their affiliated organizations, or those of the publisher, the editors and the reviewers. Any product that may be evaluated in this article, or claim that may be made by its manufacturer, is not guaranteed or endorsed by the publisher.

## Footnotes

1
https://www.w3.org/RDF/

2
https://www.dbpedia.org/

3
https://www.w3.org/TR/rdf-sparql-query/

4
https://graphql.org/

5
https://www.djangoproject.com/

6
https://graphene-python.org/

7
https://colab.research.google.com

8
https://tomsbiolab.com/vitviz

9
http://linkedlifedata.com/

10
https://sparql.uniprot.org/
