# Supplementary material for: A COMPASS for VESPUCCI: A FAIR Way to Explore the Grapevine Transcriptomic Landscape
Source: Front Plant Sci. 2022 Feb 24;13:815443. doi: 10.3389/fpls.2022.815443 (PMC8908374; doi:10.3389/fpls.2022.815443)
Supplement: Supplementary file 1 [file Table_1.docx]

A COMPASS for VESPUCCI: a FAIR way to explore the grapevine transcriptomic landscape

**Marco Moretto^1*^, Paolo Sonego^1^, Stefania Pilati^2^, José Tomás Matus^3^, Laura Costantini^4^, Giulia Malacarne^2^, Kristof Engelen^1^**

^1^Unit of Computational Biology, Research and Innovation Centre, Fondazione Edmund Mach Via E. Mach 1, 38010 San Michele all'Adige, Italy

^2^Unit of Plant Biology and Physiology, Research and Innovation Centre, Fondazione Edmund Mach Via E. Mach 1, 38010 San Michele all'Adige, Italy

^3^Institute for Integrative Systems Biology (I2SysBio), Universitat de València-CSIC, Paterna, 46908, Valencia, Spain

^4^Unit of Grapevine Genetic and Breeding, Research and Innovation Centre, Fondazione Edmund Mach Via E. Mach 1, 38010 San Michele all'Adige, Italy

*** Correspondence:**Marco Moretto
marco.moretto@fmach.it

**Keywords: gene expression, grapevine, transcriptomics, compendium, Python, R, FAIR**

***Supplementary Material***

# Supplementary Data

## Genes involved in pollen development

In this use case, we have queried the database with a short list of genes putatively involved in grapevine pollen development with the final goal of retrieving additional genes with the same function. To establish the initial set of genes we have used the outcome of the comparison of two independent parthenocarpic somatic variants with their original seeded cultivars. This list includes transcription factors with their direct targets, pectin degrading enzymes, sporopollenin biosynthetic enzymes and transporters. After the selection of the initial set of genes to query VESPUCCI, we built a module, i.e. a subset of the whole gene expression matrix composed of gene ids as rows and condition ids as columns, by retrieving the conditions where those genes are correlated and modulated. We extended the initial module adding all flower-related samples by performing a SPARQL query using the Plant Ontology term for flower, PO_0009046. To further expand the module in the other dimension, thus adding more genes, we used the distribution of gene's correlation in the current module’s conditions and added the top ranking genes. We decided to add no more than 50 new genes with a Pearson correlation coefficient of at least 0.7. The original module and the newly expanded module have been inspected for enrichment terms on both Gene Ontology as well as Plant Ontology terms. The most evident differences between the two enrichments concern the Plant ontology term “flower” and the Gene ontology term “cell wall organization”, which are over-represented in the extended module compared to the original one. The term “flower” was expected since we expanded the original module by adding flower samples. On the other hand, the over-representation of the term “cell wall organization” is likely due to the new correlated genes added that might have a role in pollen development and in particular in pollen wall formation. In order to inspect tissue-specific expression of these genes, we switched to the TPM normalized values. We ranked genes using their TPM values and evaluated how likely it is for a gene to have a certain rank considering what its expected rank value across all conditions is. With this simple procedure we evaluated whether or not a gene is “highly expressed” in a specific tissue. In order to detect new candidate genes with a potential role in pollen development we selected those genes that are more likely to be highly expressed in flowers but not expressed in fruit (and not present in our original module). Based on the annotation of the 19 extracted genes we focused on the most interesting ones. For example, VIT_12s0059g00700 that encodes a MYB transcription factor with a function in anther and pollen development, VIT_12s0057g00370 a pectinase involved in pollen wall formation, VIT_04s0008g02020 an endo-β-1,4-D-glucanase, VIT_06s0004g03790 a respiratory burst oxidase homolog protein H, VIT_06s0004g06900 a pyruvate decarboxylase, VIT_06s0009g03150 a calcium-dependent protein kinase and VIT_18s0001g07310 a Rho GTPase-activating protein, all of which are known for playing a role in pollen tube growth in model species. Finally, VIT_07s0151g00670 contains a pollen allergen domain.

## Pectin Methyl-Esterases and biotic stress

In this second use case, the main aim is the characterization of a grapevine gene family with the specific objectives to identify the gene members showing a tissue-specific expression and modulation upon biotic stresses. In particular, the genes of interest encode Pectin Methyl-Esterases (PMEs, PF01095), enzymes involved in the de-methyl esterification of pectin in the cell wall. The genes belong to a gene family of 55 members as the one identified in the 12X.v1 version of the PN40024 reference genome^^[[1]](#footnote-1)^^. In the first step of analysis, we have queried the database with the aim to inspect tissue-specific expression of these genes, using the TPM normalized values. We have searched for samples coming from 5 main different tissues (root, stem, leaf, flower and fruit) and checked gene expression levels. Samples were collected using specific Plant ontology terms used to annotate the different tissues. We ranked genes using their TPM values and evaluated how likely it is for a gene to have a certain rank considering what its expected rank value across all conditions is. With this simple procedure we evaluated whether or not a gene is “highly expressed” in a specific tissue. Then, we further performed a SPARQL query using the NCBI Taxon Ontology for pathogen, NCIT_C19157, in order to select co-expressed genes in a specific tissue and in a specific condition. This second step of analysis was based on LIMMA normalization. By this analysis, we obtained five different modules corresponding to the genes whose expression is significantly modulated upon biotic stresses and is specific for one of the five queried tissues. Our attention was principally focused on the “fruit module” because we were principally interested in looking at the PME genes potentially involved in the pectin modification during the interaction of grapevine berry with pathogens. To further expand the module in the other dimension, thus adding more genes, we used the distribution of gene's correlation in the berry module’s conditions and added the top ranking genes. We decided to add new genes with a Pearson correlation coefficient of at least 0.7. The newly expanded fruit module has been inspected for enrichment on Gene Ontology terms and it's very interesting that categories related to cell wall modification and organization and carbohydrate metabolism resulted significantly enriched, being PMEs involved in the cell-wall modification and being cell-wall a network of polysaccharide chains. We finally searched for the annotation of the genes added to the fruit module by querying the UNIPROT database using the SPARQL endpoint. We focused our attention to the most interesting genes added to the module: two cellulose synthase genes involved in the synthesis of cellulose, one of the main components of the cell wall, a Fn3_like domain-containing protein, an enzyme related to hemicellulose catabolism in the cell wall, and a PMR5N domain-containing protein, an acyl esterase involved in the regulation of carbohydrate acylation in the cell wall affecting also the resistance to pathogens.

## MYB14 transcription factor modulated genes

This use case starts with two lists of genes bound by the MYB14 transcription factor, obtained by DAP-Seq, thus representing potential direct targets. The lists are composed of thousands of genes whose MYB14-binding sites are i) positioned within -3kb (promoter binding; list 1) from their Transcription Start Site (TSS) or ii) present in the gene body up to 2kb after the end of each gene feature (i.e.; gene body and downstream binding; list 2). Once the two lists are loaded from files, we select samples coming from 5 different tissues: root, stem, leaf, flower and fruit. Similarly to what we did in the other use cases we rank genes using their TPM values and evaluate how likely it is for a gene to have a certain rank considering what its expected rank value across all conditions is. In order to deal with the high number of genes and the multiple comparison problem we calculate p-values and correct them using the Bonferroni correction since it doesn’t require any assumption on genes. To get p-values we fit a Gaussian distribution on the distribution of rank means (that will approximate a normal distribution for the central limit theorem). We now have two lists of genes (-3kb and 2kb) for each tissue and we are ready to perform a Gene Ontology Enrichment Analysis on all these sets. VESPUCCI provides a basic enrichment tool, but to perform a more complete analysis we will rely on an external package such as goatools. The enrichment analysis resulted in different GO categories associated with highly expressed, tissue-specific genes such as “aromatic amino acids family biosynthetic process” in root and “regulation of cell division” in fruit. As the last step of the analysis, we focused on gene description by retrieving the annotation from the UNIPROT database using the SPARQL endpoint highlighting once again the power of interactive environment and programmatic interfaces to build interoperable and reproducible analysis workflows.

# Supplementary Figures and Tables

## *Supplementary table 1*

## *A selection of few example queries using GraphQL, pyCOMPASS and rCOMPASS*

The following are some examples as available of queries that can be performed on VESPUCCI using the COMPASS (GraphQL), pyCOMPASS (Python) and rCOMPASS (R) interfaces. More examples are available in the documentation websites at: [https://compass-.readthedocs.io](about:blank), <https://pycompass.readthedocs.io>, and <https://onertipaday.github.io/rcompass> respectively.

| **Query** | **COMPASS (GraphQL)** | **pyCOMPASS (Python)** | **rCOMPASS (R)** |
| --- | --- | --- | --- |
| Describe all the available compendia | {  compendia {  name,  fullName,  description,  defaultVersion  versions {  versionNumber,  versionAlias,  defaultDatabase,  databases {  name,  normalizations  }  }  }  } | connect = Connect('http://compass.fmach.it/graphql') connect.describe_compendia() | get_available_compendia() |
| Get platforms information | {  platforms(compendium:"vespucci") {  edges {  node {  platformAccessId,  platformName,  description,  dataSource {  sourceName  },  platformType {  name  }  }  }  }  } | compendium = connect.get_compendium('vespucci') Platform.using(compendium).get() | get_platform_information(compendium = "vespucci") |
| Get experiment information | {  experiments(compendium:"vespucci") {  edges {  node {  organism,  experimentAccessId,  experimentName  }  }  }  } | compendium = connect.get_compendium('vespucci') Experiment.using(compendium).get() | get_experiments(compendium = "vespucci") |
| Get sample sets by sample IDs | {  sampleSets(compendium:"vespucci", samples:["U2FtcGxlVHlwZTox"]) {  edges {  node {  id,  name  }  }  }  } | samples = Sample.using(compendium).get(filter={‘id’:’U2FtcGxlVHlwZTox’})  SampleSet.using(compendium).by(samples=samples) | get_sampleset_by_sampleid(compendium = "vespucci", samples =”U2FtcGxlVHlwZTox”, normalization=”limma”) |
| Get sample annotation triples | {  annotationPrettyPrint(compendium:"vespucci", ids:"U2FtcGxlVHlwZTox") {  rdfTriples  }  } | compendium = connect.get_compendium('vespucci') sample = Sample.using(compendium).get(filter={‘id’:’U2FtcGxlVHlwZTox’}) Annotation(sample).get_triples() | get_annotation_triples(compendium = "vespucci", ids = “U2FtcGxlVHlwZTox”) |
| Get genes by names | {  biofeatures(compendium:"vespucci", name:"VIT_00s0332g00060") {  edges {  node {  name,  biofeaturevaluesSet(bioFeatureField_Name:"sequence") {  edges {  node {  value  }  }  }  }  }  }  } | compendium = connect.get_compendium('vespucci')  BiologicalFeature.using(compendium).get(filter={‘name’: "VIT_00s0332g00060"}) | get_biofeature_by_name(compendium = "vespucci", name_In = c("VIT_00s0332g00060")) |
| Create a module with biological features and sample sets | {  modules(compendium: "vespucci", version:"legacy", biofeaturesIds: ["QmlvRmVhdHVyZVR5cGU6MQ==","QmlvRmVhdHVyZVR5cGU6Mg=="], samplesetIds: ["U2FtcGxlU2V0VHlwZToxMjYw", "U2FtcGxlU2V0VHlwZToxMjYx", "U2FtcGxlU2V0VHlwZToxMjYy"]) {  normalizedValues  sampleSets {  edges {  node {  id  name  normalizationdesignsampleSet {  edges {  node {  sample {  sampleName  }  }  }  }  }  }  }  biofeatures {  edges {  node {  id  name  }  }  }  }  } | bf = BiologicalFeature.using(compendium).get(filter={‘id_In’: ["QmlvRmVhdHVyZVR5cGU6MQ==","QmlvRmVhdHVyZVR5cGU6Mg=="]})  ss = SampleSet.using(compendium).get(filter={‘id_In’: ["U2FtcGxlU2V0VHlwZToxMjYw", "U2FtcGxlU2V0VHlwZToxMjYx", "U2FtcGxlU2V0VHlwZToxMjYy"]})  Module.using(compendium).create(biofeatures=bf, samplesets=ss) | ss <- c("U2FtcGxlU2V0VHlwZToxMjYw", "U2FtcGxlU2V0VHlwZToxMjYx", "U2FtcGxlU2V0VHlwZToxMjYy")  bfs <- c("QmlvRmVhdHVyZVR5cGU6MQ==","QmlvRmVhdHVyZVR5cGU6Mg==")  create_module(biofeaturesNames = bfs, samplesetNames = ss,  normalization = "legacy", useIds = TRUE) |

### *Supplementary Table 2*

*Comparison table between VESPUCCI v1 and v2 main features*

|  |  | **V1** | **V2** |
| --- | --- | --- | --- |
| **Data content** | **Number of microarray samples** | 1605 | 3682 |
|  | **Number of RNA-seq samples** | 139 | 3598 |
| **Interface** | **GUI** | Yes | Yes |
|  | **Python interface** | No | Yes |
|  | **R interface** | Basic functionalities | Yes |
| **Sample annotation** | | Structured with an in-house developed controlled vocabulary | Structured using standard ontologies and RDF data model |
| **Data normalization** | | Sample contrast (logratio between one reference and one test sample) | Sample contrast: logratio between one reference and one test sample (for V1 data only) |
|  |  |  | Condition contrast: logratio between a reference and a test condition using LIMMA. A condition is represented by one or more replicated samples |
|  |  |  | TPM: Transcript Per Million (for RNA-seq data only) |

### *.*

### *Supplementary Table 3*

### *Comparison table between several on-line resources for grapevine and other plant species.*

| **Resource name** | **Organism** | **Resource type** | **Data type** | **Access type** | **Resource availability** | **Code availabillity** | **Website** |
| --- | --- | --- | --- | --- | --- | --- | --- |
| VESPUCCI v2 | Grapevine | Database and tools | Gene expression (RNA-seq and microarray) | Programmatic / GUI | Free | Open source | http://compass.fmach.it/vespucci |
| TomExpress | Tomato | Database and tools | Gene expression (RNA-seq) | GUI | Free | Closed source | http://tomexpress.toulouse.inra.fr |
| Genevestigator | Arabidospis | Database and tools | Gene expression (RNA-seq and microarray) | Programmatic / GUI | Commercial | Closed source | https://genevestigator.com |
| VitisCyc | Grapevine | Database | Metabolic pathway | GUI | Free | Closed source | http://pathways.cgrb.oregonstate.edu |
| VTCdb | Grapevine | Database and tools | Co-expression network | GUI | Free | Closed source | http://vtcdb.adelaide.edu.au |
| VTC-Agg | Grapevine | Database | Co-expression network | GUI | Free | Closed source | https://sites.google.com |
| Vitis OneGenE | Grapevine | Tool | Association network | GUI | Free | Open source | http://vitis.onegenexp.eu |
| Vitis Visualization Platform (VitViz) | Grapevine | Tool | Gene expression atlases, aggregated co-expression networks, DAP-Seq data integrated to Genome Browsers | GUI | Free | Not applicable | https://tomsbiolab.com/vitviz |
| Grape eFP Browser | Grapevine | Tool | Gene expression (RNA-seq) | GUI | Free | Closed source | http://bar.utoronto.ca/efp_grape/cgi-bin/efpWeb.cgi |
| miRVIT | Grapevine | Database | miRNA expression (RNA-seq) | GUI | Free | Closed source | http://mirvit.ipsp.cnr.it |
| Grape-RNA | Grapevine | Database and tools | Gene expression (RNA-seq) | GUI | Free | Closed source | http://www.grapeworld.cn/gt/ |
| BIOWINE | Grapevine | Database and tools | Gene expression (RNA-seq) | GUI | Free | Closed source | https://alpha.dmi.unict.it/biowine/ |
| VitisNet | Grapevine | Database | Metabolic pathway | GUI | Free | Not applicable | http://vitis-dormancy.sdstate.org/pathways.cfm |

###

## Supplementary Figures


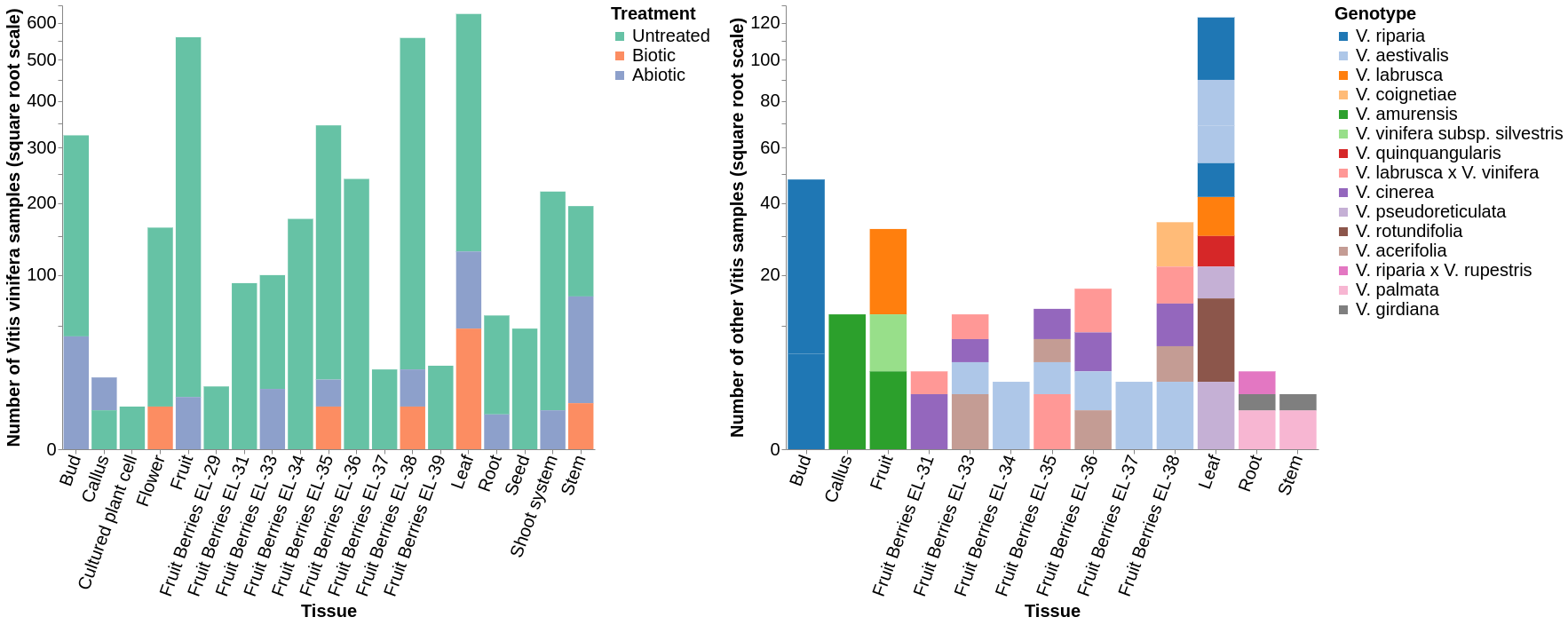


**Supplementary Figure 1.** Bar Plot of VESPUCCI v1 sample distributions based on their condition annotation in Vitis vinifera (left) and non-vinifera (right) experiments. Samples are divided by tissues (X-axis) and their abundances in square root scale (Y-axis). In the left plot, different colors are used to denote untreated samples, biotic-treated samples and abiotic-treated samples. In the right plot, colors are used to differentiate between different Vitis species, subspecies and cross species. The great majority of samples (50%) come from Vitis vinifera untreated fruit samples taken at different developmental stages. Non-vinifera species and hybrids samples represent 7.5% of the dataset while 8.5% of the total are stress-related (1.4% being fruit) and 34% are Vitis vinifera untreated non-fruit samples.

1. <http://plants.ensembl.org/Vitis_vinifera/Info/Index> [↑](#footnote-ref-1)
